# Perspectives on the Role of Thoracic Fascial Blocks in Cardiac Anaesthesia: Will They Represent a New Era?

**DOI:** 10.3390/jcm14030973

**Published:** 2025-02-03

**Authors:** Giuseppe Sepolvere, Daniele Marianello, Cristina Santonocito, Simone Messina, Simona Silvetti, Federico Franchi, Gianluca Paternoster, Filippo Sanfilippo

**Affiliations:** 1 Intensive Care Unit, Department of Anesthesia and Cardiac Surgery, San Michele Hospital, 81024 Caserta, Italy; 2 Cardiothoracic and Vascular Anesthesia and Intensive Care Unit, Department of Medical Science, Surgery, and Neurosciences, University Hospital of Siena, 53100 Siena, Italy; d.marianello83@gmail.com (D.M.); federico.franchi@unisi.it (F.F.); 3 Department of Anesthesia and Intensive Care, University Hospital “Policlinico-San Marco”, 24046 Catania, Italy; cristina.santonocito@gmail.com (C.S.); messina.simone05@gmail.com (S.M.); 4 Department of Cardiac Anesthesia and Intensive Care, Cardiovascular Network, IRCCS Policlinico San Martino Hospital, 16132 Genoa, Italy; lu.simo@hotmail.it; 5 Department of Health Science, Anesthesia and ICU, School of Medicine, University of Basilicata San Carlo Hospital, 85100 Potenza, Italy; paternostergianluca@gmail.com; 6 Department of General Surgery and Medico-Surgical Specialties, School of Anesthesia and Intensive Care, University of Catania, 95124 Catania, Italy

**Keywords:** cardiac surgery, opioid-sparing, enhanced recovery, multimodal analgesia, regional anaesthesia, peripheral nerve block

## Abstract

Cardiac surgery is continuously evolving, with increasing skills required by the cardiac anaesthesiologist. Following the advent of intraoperative echocardiography, we are witnessing a potential new revolution for the cardiac anaesthesiologist. A new era has indeed started with the implementation of thoracic fascial blocks (TFBs) in the field of cardiac surgery. TFBs provide several advantages in the context of multimodal analgesia, with improved pain control and reduction of the side effects related to large doses of opioids. We envisage that implementation of TFBs is likely to become a pivotal concept in the field of enhanced recovery after cardiac surgery. We describe the main TFBs for the anterior and/or antero-lateral chest wall, and their peculiar use in cardiac surgery. In particular, we discuss indications and tips and tricks to enhance clinical results for the following blocks: (1) Pecto-Intercostal Plane (superficial and deep); (2) Rectus Sheath; (3) Interpectoral Plane and Pectoserratus Plane; (4) Serratus Anterior Plane; (5) Erector Spinae Plane. Nonetheless, the scientific evidence for the use of TFBs in the field of cardiac anaesthesia is not robust yet, mostly based on small-sized single-centre studies, making it difficult to achieve a high quality of evidence. Further, it remains unclear which cardiac surgery patients may benefit the most from these techniques.

## 1. Introduction

Several peculiarities characterize the setting of cardiac anaesthesia. Among others, the complexity of patients and procedures, the use of cardiopulmonary bypass and the complications associated with cardiac surgery [1,2,3], and the advanced haemodynamic skills and the knowledge of echocardiography [4,5,6,7] make this branch of the anaesthesiological discipline among the most challenging ones. Interestingly, from decade to decade, turnarounds have happened. For instance, the use of large doses of opioids emerged as a very useful analgesic strategy in the 1990s [8,9,10]; in contrast, current evidence suggests an opioid-sparing approach for cardiac anaesthesia, with several possible advantages. Among these, a lower dose of opioids may facilitate postoperative recovery, boosting the subsequent rehabilitation process, and may reduce the risk of postoperative opioid-induced hyperalgesia [11,12]. Indeed, multimodal analgesia with opioid stewardship and early postoperative ambulation and upper extremity exercise were among the approaches introduced in the recent Expert Consensus Statement on Perioperative Care in Cardiac Surgery [13]. Another turnaround in cardiac surgery has been the exponential increase in the use of transoesophageal echocardiography, now widely recognized as a standard skill for up-to-date cardiac anaesthesiologists. Further, the cardiac surgery discipline had introduced approaches and techniques to reduce complications and facilitate the patient’s recovery. In this case, the introduction of the miniaturized cardiopulmonary bypass and of mini-invasive surgical approaches could be regarded as manifestos. In the context of enhanced recovery after cardiac surgery (ERACS), so-called “fast track extubation” (postoperative extubation achieved within 6 h) was promoted at the beginning of the twenty-first century [14]. To achieve the goal, several criteria must be present (i.e., steady haemodynamic and respiratory conditions, absence of bleeding, good metabolic profile, etc). Among these, good analgesic control is crucial. Analgesia had previously been achieved with a multimodal approach based on opioid infusion, especially during the first 24–48 h. Nowadays, there is a different analgesic approach to the cardiac surgery patient. Undeniably, there has been an exponential increase in the use of Thoracic Fascial Blocks (TFBs) with consequent improvements in the quality of pain management and in the reduction of complications due to opioid-based regimens, such as the risk of opioid-induced hyperalgesia as well as chronic pain syndrome [15,16], associated particularly with the use of remifentanil [17]. The adoption of TFBs (also known as fascial plane nerve blocks) has the potential to reduce acute postoperative pain whilst simultaneously decreasing the perioperative consumption of opioids. Hence, TFBs are perfectly aligned with the ERCAS philosophy, though the guidelines published in 2019 [18] still did not include them in their recommendations. Conversely, the above-mentioned recent consensus statement published in 2024 [13] also included techniques of peripheral regional anaesthesia, with a moderate level of evidence, confirming the growing interest in the field. Moreover, a joint consensus of the PeriOperative Quality Initiative and the ERACS Society regarding pain management and opioid stewardship in adult cardiac surgery strongly encourages opioid stewardship and multimodal analgesia (level of evidence B for both), whilst the recommendation to consider regional techniques is categorized as weak and of level C.

In this manuscript, we provide a summary of the TFBs of potential interest in cardiac surgery procedures, with indications and practical suggestions for each of them, along with a discussion on the evidence in their support and on the current knowledge gap, which explain the weakness of the current recommendations.

## 2. Thoracic Fascial Blocks in Cardiac Surgery

TFBs and, in particular, the anterior chest wall blocks encompass the antero-medial and antero-lateral thoracic wall. Conversely, the posterior chest wall blocks target the posterior thoracic wall and are widely utilized across various surgical fields [19].

The anterior and antero-lateral chest wall blocks are of interest in the field of cardiac surgery and, according to a recent consensus standardizing their nomenclature [20], they include the following:

- Pecto-Intercostal Plane (PIP) Block;
- Rectus Sheath Block (RSB);
- Interpectoral Plane (IPP) Block;
- Pectoserratus Plane (PSP) Block;
- Serratus Anterior Plane (SAP) Block;
- Erector Spinae Plane (ESP).

In this part of the manuscript, we describe the main techniques and advantages of the above-mentioned blocks in reference to the cardiac surgery setting. In Table 1, we list the main indications for each block in the field of cardiac surgery, also describing the nervous and anatomical targets, the injection site, and a suggested volume of local anaesthetic (LA). Tips and tricks for a successful technique are also provided in the table. However, we do not provide suggestions on which LA (or possible combinations of these drugs) should be used as this is not the focus of the manuscript, and the evidence in the field of cardiac surgery is rather limited. In general, as a rule of thumb, if a TFB is performed with the intention to provide anaesthesia, a LA concentration of 0.375–0.5% seems appropriate [21], whilst if the target is to provide analgesia, a much lower concentration in the LA solution may be used (i.e., 0.125–0.25%). As for other regional blocks, adjuvants are useful to enhance TFBs [22]; in this regard, we suggest considering the addition of dexmedetomidine or clonidine at 0.5–1 mcg/kg (to the LA solution or intravenously), and/or the administration of dexamethasone with an intravenous dose of 2 to 4 mg [23,24,25,26,27]. Some of the side effects related to the use of TFBs could be more relevant in cardiac surgery patients. For instance, bleeding at the site of insertion could be more common due to the concomitant anticoagulant/antiplatelet therapy, and intravascular injection with associated risk of systemic toxicity may be particularly relevant in patients with cardiac disease and/or arrhythmias. Further, TFBs may expose the patient to the risk of intramuscular injection with haematoma, pleural puncture, and haematoma of internal mammary artery (IMA).

### 2.1. Pecto-Intercostal Plane (PIP) Block

The PIP block targets the anterior cutaneous branches from the T2 to the T6 intercostal nerves, innervating the anteromedial chest wall and penetrating the intercostal and pectoralis major muscles (IM and PMM, respectively). The procedure involves superficial and deep PIP blocks, depending on whether the injection is performed above or below the IM. Bilateral injection is necessary to achieve complete sternal area coverage due to significant nervous overlap. The PIP block may provide analgesia and anaesthesia for various cardiac procedures, including median sternotomy, sternal fractures, internal mammary region interventions, and insertion of an Implantable Cardioverter Defibrillator (ICD, in this case combined with the SAP block) [28,29,30,31].

#### 2.1.1. Technique for Superficial PIP Block

**Figure 1:**
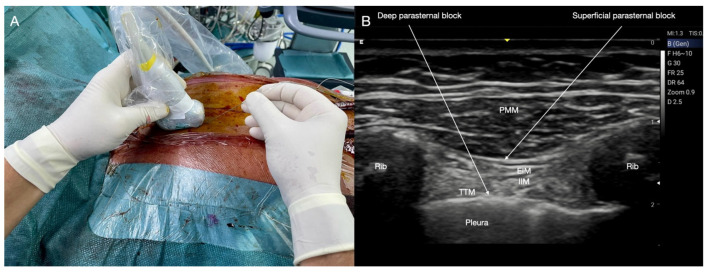
Superficial and deep Pecto-Intercostal Plane block. (**A**) Patient, probe, and needle position. (**B**) Superficial and deep parasternal block ultrasound landmarks. PMM: pectoralis major muscle; EIM: external intercostal muscle; IIM: internal intercostal muscle; TTM: transversus thoracis muscle; Rib: rib; Pleura: pleura.

The patient lies supine with arms extended alongside the body and the head in a neutral position. A high-frequency linear probe is positioned approximately 2 cm parallel and lateral to the sternum’s border, initially at the second and then at the fourth intercostal space. The needle is inserted using an in-plane approach in a cranial-to-caudal or caudal-to-cranial direction (Figure 1A). Ultrasound landmarks include the PMM and external intercostal muscle (EIM), visualization of ribs and their posterior shadow cones, and the pleura sliding (Figure 1B).

**Figure 2:**
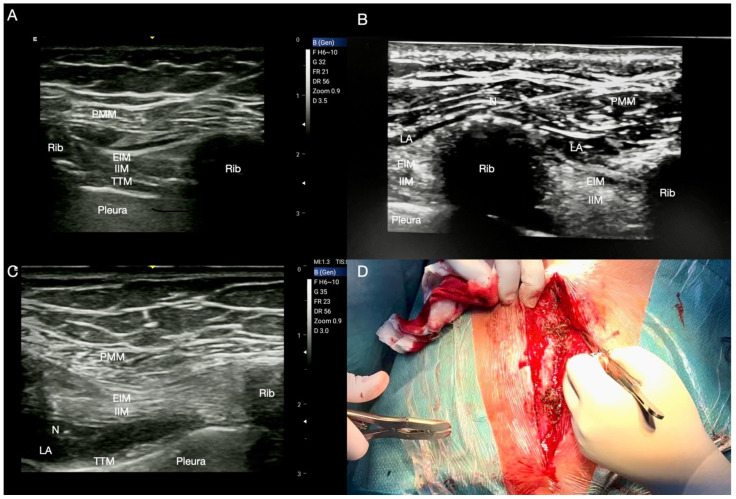
Pecto-Intercostal Plane (PIP) block. (**A**) Superficial PIP block: the local anaesthetic (LA) is injected between the pectoralis major and the external intercostal muscles (PMM and EIM, respectively); (**B**) modified superficial PIP block: the LA is injected under the PMM, placing the tip of the needle (N) on the rib dome and allowing a more homogeneous and longitudinal spread of LA; (**C**) deep PIP block: the LA is injected between the internal intercostal and the transversus thoracis muscles (IIM and TTM, respectively); (**D**) sternal wire removal.

Usually, a 5–10 mL solution of LA is injected into the fascial plane between the PMM and EIM at the second and fourth intercostal spaces (Figure 2A). Scimia et al. [32] suggested a modified approach, positioning the needle tip on the rib dome in order to reduce the LA volume and to achieve a more homogeneous and longitudinal spread into the target fascial compartment (Figure 2B). Moreover, ribs are a useful anatomical landmark in the absence of good visibility of the fascial plane and also represent a safety stop region (similar to what happens during the SAP block or with transverse process in the ESP block). The evolution of the superficial PIP block has significantly advanced in recent years. Initially proposed for managing intra- and postoperative pain from cardiac sternotomy, it could be considered for delivering anaesthesia for sternal surgery, with the advantage of avoiding general anaesthesia and the associated risks in selected patients with high load of comorbidities (Figure 2D) [33,34,35].

#### 2.1.2. Technique for Deep PIP Block

The anterior cutaneous nerves run over the deep transversus thoracis muscle (TTM) before crossing the internal and parasternal muscles near the sternal border to reach the superficial plane. This anatomical arrangement also allows the choice of a deeper injection and blockade, which was initially described as the transversus thoracis plane block [36]. Patient and probe positioning, as well as the approach and the needle insertion, are identical to the superficial block (Figure 1A). The ultrasound anatomy of the deep PIP block is depicted in Figure 1B. The LA is injected between the internal intercostal muscle (IIM) and the TTM (Figure 2C).

**Figure 3:**
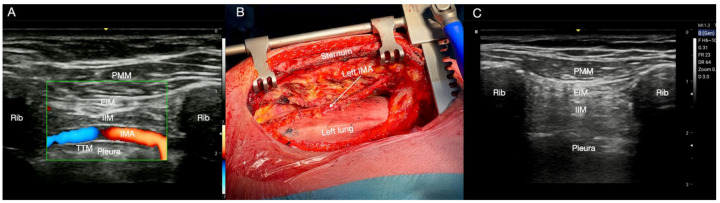
Internal mammary artery anatomy. (**A**) The internal mammary artery (IMA) runs between the internal intercostal and transversus thoracis muscles (IIM and TTM, respectively), as shown by the Doppler imaging; (**B**) IMA harvesting, (**C**) ultrasound image of the landmarks after IMA harvesting: note the TTM disruption rendering the transversus thoracis plane block difficult to perform. Pectoralis major muscle (PMM); external intercostal muscle (EIM).

Both superficial and deep PIP blocks exhibit similar analgesic and anaesthetic efficacy, although some relevant anatomical considerations should be made. It should be noted that the TTM is often small and not always visible with ultrasound. At the fourth parasternal intercostal space, the IMA and the internal mammary vein (IMV) can be identified between the IIM and the TTM as a longitudinal structure approximately 1.5 cm from the lateral border of the sternum (Figure 3A). Apart from the risk of pneumothorax, such anatomical proximity to the IMA and IMV suggests the need for caution to mitigate the risks of LA systemic toxicity, IMA injury, or haematoma (Figure 3B). Additionally, patients undergoing IMA harvesting may experience some tissue disruption in the transversus thoracis plane, complicating TTM identification and making the deep PIP block impractical (Figure 3C). Further, when performing the block before cardiac surgery, the potential damage to the right and left IMAs would make these targets unsuitable for bypass grafting. Considering these factors, when compared to the deep PIP block, a superficial approach may be considered safer, still allowing satisfactory analgesia whilst safeguarding the territory of the IMA as it is performed farther from the vascular structures, pleura, and the heart [37].

### 2.2. Rectus Sheath Block

Although the Rectus Sheath Block (RSB) is categorized as an abdominal wall block, it provides comprehensive analgesia to the anterior chest wall when combined with the parasternal block. RSB offers somatic analgesia to the anteromedial abdominal wall and periumbilical area following midline laparotomy for upper abdominal surgery by targeting the anterior cutaneous branches from T9 to T12 of the intercostal nerves [38,39,40]. The introduction of ultrasound has transformed the RSB into a safe and effective procedure in the context of Enhanced Recovery After Surgery (hence possibly for ERACS too), reducing opioid requirements, and mitigating the risks associated with neuraxial techniques and the hazards of vascular injury. In addition to pain due to median sternotomy, the insertion of chest drains also represents a significant source of discomfort after cardiac surgery. Among other sources, such pain may stem from skin incision, ongoing irritation of adjacent tissues, and direct injury to the rectus abdominis muscles from the chest drains. Effective pain control at the insertion sites is very useful in pain management after cardiac surgery. Severe postoperative pain in the epigastric region compromises the patient’s respiratory dynamics, increasing the risk of respiratory complications, and may also delay extubation [41,42].

The RSB is typically performed at the conclusion of cardiac surgery following the placement of subxiphoid chest drains and can significantly reduce discomfort and pain arising from this area. Whilst the infiltration of the chest drain insertion site with LA may represent a faster and easier option, a randomized controlled trial (RCT) showed that in patients undergoing cardiac surgery with chest drains inserted from the epigastric region and receiving an IPP block, the addition of RSB improved analgesia as compared to local infiltration with LA [43]. The RSB is now an integral part of an effective multimodal opioid-sparing approach, often combined with superficial or deep PIP blocks, to manage pain stemming from median sternotomy and subxiphoid drainages, in both paediatric and adult populations [44,45].

#### Technique for RSB

**Figure 4:**
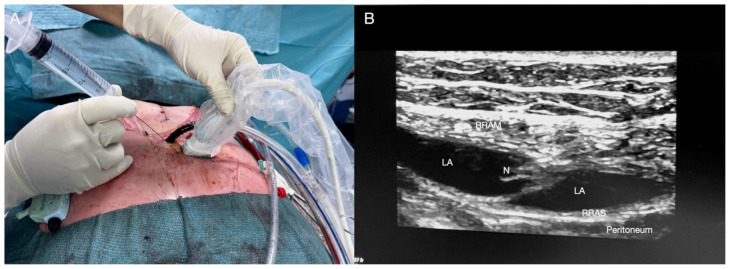
Rectus sheath block. (**A**) Patient, probe, and needle position. (**B**) The local anaesthetic (LA) is deposited between the rectus muscle and its sheath. RRAM: right rectus abdominis muscle; RRAS: right rectus abdominis sheath; N: needle; Peritoneum: peritoneum.

With the patient in the supine position, a high-frequency linear probe is positioned just below the tube emergence from the skin (Figure 4A). Ultrasound landmarks include the skin, the right and left rectus abdominis muscles (RAM and LAM, respectively), the rectus abdominis muscle sheath (RAMS), and the peritoneum. Using an infraumbilical-pararectus approach and bilateral injection (from lateral to medial followed by medial to lateral), a volume of 10–15 mL of LA is deposited between the rectus muscle and its sheath (Figure 4B).

### 2.3. Interpectoral Plane Block (IPP) and Pectoserratus Plane Block (PSP)

The IPP targets the lateral and medial pectoral nerves (LPN and MPN, respectively), innervating the PMM and pectoralis minor (pmm) muscles. The LPN is typically proximate to the pectoral branch of the thoraco-acromial artery (TAA), used as a vascular landmark for the IPP. Blanco introduced a modified version, known as PSP, which extends the block on the lateral branches of the intercostal nerves from T2 to T6 and to the axillary region. From a practical standpoint, the PSP offers no significant advantages as compared to the IPP in the cardiac surgery setting as the extension of the block itself to the axilla does not add much pain relief in the context of most cardiac surgical procedures. Moreover, PSP may result in some dispersion of the LA solution. Hence, a more reasonable option seems the combination of IPP with a SAP block [46,47].

#### Technique for IPP and PSP

**Figure 5:**
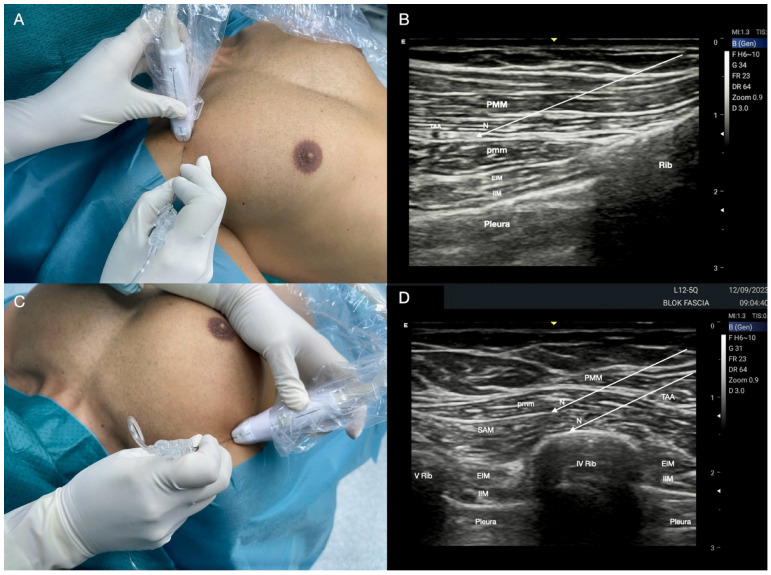
Interpectoral plane (IPP) and Pectoserratus Plane (PSP) blocks. (**A**) IPP block, patient and probe position. The patient is placed in the supine position with the head rotated in the opposite direction from the injection site, exposing the deltoid-pectoral groove. A linear high-frequency probe is positioned in the deltoid-pectoral groove. The needle is inserted with the in-plane approach in a medial-to-lateral direction or in a lateral-to-medial direction. (**B**) IPP block, ultrasound landmarks and local anaesthetic injection. From top to bottom: pectoralis major muscle, pectoralis minor muscle, pectoral branch of the thoracoacromial artery, rib, external and internal intercostal muscles, pleura. The local anaesthetic is injected between the pectoralis major and minor muscles following the pectoral branch of the thoracoacromial artery. (**C**) PSP block, patient and probe position. The probe slides towards the axilla at the level of the fourth rib. The needle is inserted with the in-plane approach in a medial-to-lateral direction. (**D**) PSP block, ultrasound landmarks and local anaesthetic injection. Local anaesthetic is injected between the pectoralis minor and the serratus anterior muscles or between the serratus anterior muscle and the intercostal muscle at the level of the fourth rib. PMM: pectoralis major muscle; pmm: pectoralis minor muscle; SAM: serratus anterior muscle; IIM: internal intercostal muscle; EIM: external intercostal muscles; TAA: pectoral branch of the thoraco-acromial artery; Rib: rib; Pleura: pleura; N: needle.

**IPP**: the patient is positioned supine with the head rotated contralaterally, exposing the deltoid-pectoral groove opposite to the injection site. Using a linear high-frequency probe positioned in the groove, the needle is inserted with an in-plane approach from a medial-to-lateral direction. Alternatively, a lateral-to-medial approach may be chosen for greater patient and practitioner comfort (Figure 5A). Ultrasound landmarks include the PMM, the pmm, the TAA, and the rib (Figure 5B). Following TAA visualization, usually a 10 mL volume of LA is injected into the fascial plane between the PMM and the pmm (Figure 5B).

**PSP:** the patient and the probe positions are similar to the IPP block. By tilting the probe medially towards the thorax, the axillary artery and vein are identified, with the second rib visualized beneath these vessels. Moving the probe distally and laterally toward the axilla, the lateral margin of the PMM is located beneath, and lies between the third and the fourth rib. Near the latter, landmarks such as the ligament of Gerdy, indicating the axillary cavity entrance, along with the pmm and the serratus anterior muscle (SAM), are identified (Figure 5C). Sono-anatomy includes PMM, pmm, SAM, IM, ribs, and pleural sliding (Figure 5D). Following injection of LA for achieving the IPP block, a PSP requires an additional 20 mL volume of LA between the pmm and the SAM, or optionally between the SAM and the IM at the level of the fourth rib (Figure 5D).

### 2.4. Serratus Anterior Plane Block

Introduced by Blanco as a modification of the IPP and PSP blocks, the Serratus anterior plane (SAP) block targets the lateral intercostal nerves from T2 to T6–T9, providing analgesia for a vast portion of the hemithorax [48]. Indications range from breast cancer and thoracic surgery to cardiac procedures like mitral valve surgery in mini-thoracotomy. Some authors have suggested single-shot D-SAP block with dexmedetomidine for effective postoperative pain control after mini-thoracotomy heart surgery [49]. For the implant of ICD or pacemaker, a SAP block should be combined with IPP or PIP. Other indications are listed in the Table 1.

#### Technique for SAP Block

**Figure 6:**
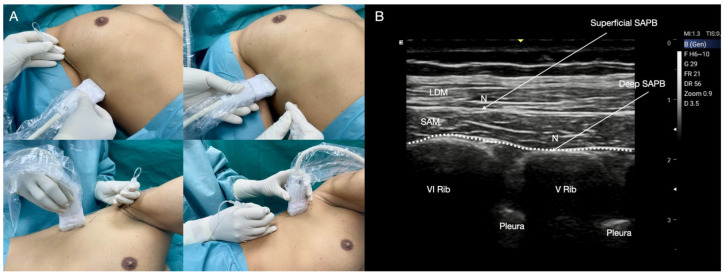
Serratus anterior plane (SAP) block. (**A**) Probe, patient position, and cranial-to-caudal or caudal-to-cranial approach. (**B**) SAP block landmarks and local anaesthetic (LA) injection. From the top: latissimus dorsi muscle (LDM), serratus anterior muscle, ribs, and pleura. The superficial SAP block is performed by the injection of LA between the LDM and serratus anterior muscle (SAM, above the surface of serratus anterior muscle), while the deep SAP block is performed by the injection of LA between the SAM and the fifth rib (below the surface of the SAM). The continuous infusion catheter is placed between the anterior muscle and the ribs (white dotted line). Rib: rib; Pl: pleura; N: needle: Catheter: white dotted line.

The patient may assume a supine or lateral position (Figure 6A). Ultrasound landmarks include the Latissimus Dorsi muscle (LDM), SAM, ribs, and pleura (Figure 6B). Thoracodorsal artery (TDA) visualization allows the identification of the SAM superficial plane. The SAP block can be superficial (S-SAP) or deep (D-SAP), depending on whether the LA is injected above or below the SAM. The S-SAP block usually requires the injection of a 30 mL volume of LA between the LDM and the SAM, while the D-SAP block entails injecting a similar LA volume between the SAM and the fifth rib (Figure 6B). Notably, the D-SAP block target area aligns with the PSP block below the SAM, making them anatomically similar. The plane of the SAM is also anatomically suitable for the placement of a catheter in order to manage both intra- and postoperative pain through the continuous infusion of LA in patient-controlled analgesia [30] (Figure 6B). Notably, the catheter could also be inserted between the LDM and the SAM. However, in our opinion, insertion between the SAM and the ribs may simplify the technique, also conferring greater stability to the catheter with lower risk of displacement.

### 2.5. Erector Spinae Plane Block

The erector spinae plane (ESP) block was first described by Forero et al. in 2016 as a novel analgesic procedure to manage neuropathic pain resulting from metastatic disease of the ribs or from multiple rib fractures, providing an extensive dermatomal sensory block [50]. Indications for the ESP block include a wide range of surgical procedures, including breast surgery, cardiac and thoracic surgery, abdominal surgery, urological surgery, orthopaedic, and spine surgery. The block is now increasingly performed across various surgical fields due to its safety and simplicity as compared to established thoracic paravertebral or epidural blocks, which carry additional risks related to anticoagulation and/or platelet inhibition in the field of cardiac surgery [51].

The mechanism of the ESP block involves the spread of LA close to or directly into the paravertebral space, where the ventral and dorsal rami of the spinal nerves emerge and then diverge. The ESP block site also allows catheter insertion for continuous infusion of LA, providing lasting intra- and postoperative analgesia, especially for pain relief after mini-thoracotomy in the context of cardiac surgery. It is believed to relieve pain in a large area of the posterior, lateral, and anterior chest and lumbar walls, also preventing visceral pain. It can be used for acute and chronic pain of the trunk, as well as for upper and lower limbs. However, it may not be as effective for managing pain from midline sternotomy, as demonstrated in a systematic review and meta-analysis by King et al. [51]. In support of these findings, Dost et al. demonstrated the greater efficacy of a combined superficial PIP block associated with ESP as compared to a bilateral ESP block alone in the management of acute post-sternotomy pain [52,53].

#### Technique for ESP Block

**Figure 7:**
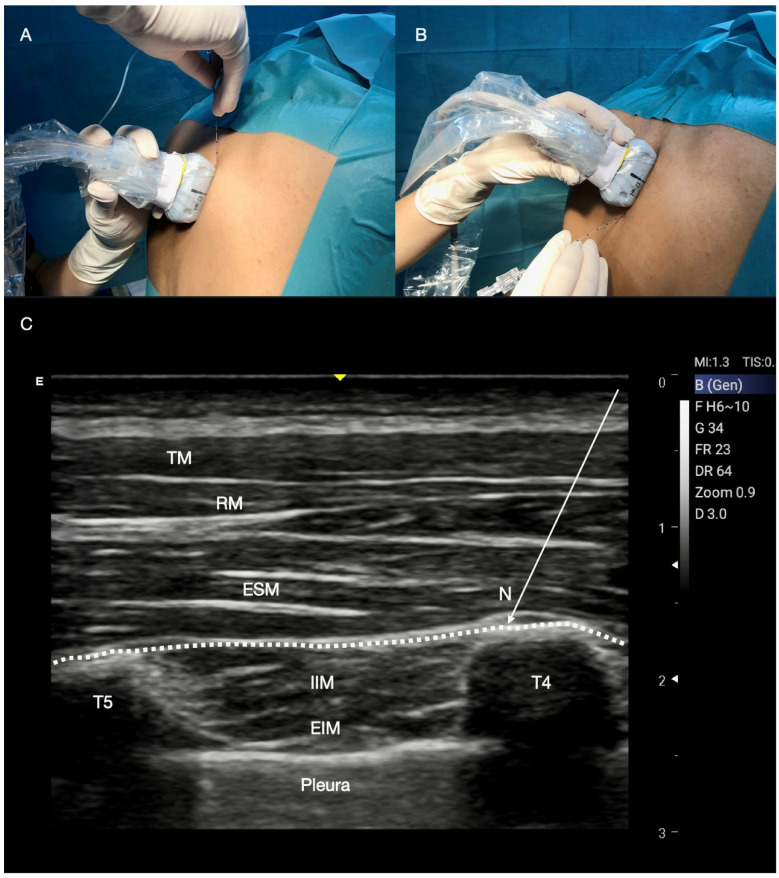
Erector Spinae Plane (ESP) block. (**A**,**B**) Patient, probe position, and needle insertion. The patient may assume the seated, prone, or lateral position. A high-frequency linear probe is placed approximately 3 cm lateral to the interspinous line at the level of the T3–T4 or T5–T6 transverse processes. The needle is inserted with the in-plane approach in a cranial-to-caudal direction (**A**) or in a caudal-to-cranial direction (**B**). (**C**) ESP block, ultrasound landmarks and local anaesthetic injection. From top to bottom: trapezius, rhomboid, erector spinae, internal intercostal and external intercostal muscles, transverse processes, pleura. The local anaesthetic is injected between the transverse process and the erector spinae muscle (ESM). TM: trapezius muscle; RM: rhomboid muscle; T4: T4 transverse process; T5 transverse process; IIM: internal intercostal muscle; EIM: external intercostal muscles; Catheter: white dotted line.

The block is typically performed with the patient in the seated, prone, or lateral position, using ultrasound guidance to identify landmarks and administer LA. A high-frequency linear probe is placed approximately 3 cm lateral to the interspinous line at the level of the T3–T4 or T5–T6 transverse processes (Figure 7A,B). In the paraspinal area, the correct landmarks are trapezius, rhomboid, erector spinae muscles, transverse processes, and pleura. The needle is inserted through an in-plane approach in a cranial-to-caudal (or vice versa, from caudal-to-cranial) direction, and usually a volume of 20–25 mL of LA is injected into the plane between the edge of the transverse process (TP) and the erector spinae muscle (ESM) (Figure 7C).

It is most likely that the injected LA spreads through the costotransverse foramen and several slits of the superior costotransverse ligament (SCTL), which represents the posterior wall of the paravertebral space [54,55,56]. On this basis, several techniques deriving from the intertransverse process (ITP) blocks have been introduced under different nomenclatures with the aim to inject the LA a little closer to the slits of the SCTL than the ESP block, in order to obtain a more effective diffusion of the LA to the paravertebral space. The plane between the ESM and the TPs represents an optimal anatomical site where a catheter may be inserted to perform continuous infusion of LA, and provide lasting intra- and postoperative analgesia in cardiac surgery [57], especially for pain deriving from mini-thoracotomy [58] (Figure 7C). The catheter may be inserted with the patient in a seated position; however, it is preferable to perform the continuous ESP block before induction of general anaesthesia with the patient in the lateral decubitus to minimize the haemodynamic instability that may occur when placing cardiac patients in a sitting position. The alternative is represented by the continuous D-SAP block performed before induction or at the end of surgery with the patient already in a supine position. The ESP block tips and tricks are summarized in Table 1.

## 3. Thoracic Fascial Blocks in Cardiac Surgery: What Is the Evidence?

Regional anaesthesia with neuroaxial blocks (epidural, spinal) in cardiac surgery patients have been studied for decades [59,60]. However, the risk of epidural haematoma, which ranges from 1:1000 to 1:3000 in cardiac surgery cases, has significantly discouraged such practice [61,62]. In truth, a meta-analysis of 51 RCTs enrolling over 4000 patients suggested that thoracic epidural anaesthesia reduced the intensive care unit and hospital length of stay, with minimal incidence of epidural haematomas [63]. Nonetheless, this practice currently seems rather uncommon, and almost abandoned, as most patients are now treated with antiplatelets and/or anticoagulants, with inherent risks of bleeding/haematoma formation.

More importantly, new approaches for the management of postoperative analgesia in patients undergoing cardiac surgery are currently available. In light of the relative ease of performing the previously described TFBs (especially when an ultrasound technique is implemented), the very low risks of complications (which are of minor impact in most cases), and the importance of boosting the patient’s recovery in the context of ERACS and reducing healthcare costs, it seems rather obvious that TFBs represent an important advancement in the setting of cardiac surgery. However, the current recommendations in favour of TFBs are weak and categorized with level of evidence C. This could be the result of a currently growing but still insufficient number of RCTs, which does not allow us to draw firm conclusions. Moreover, interpretation is limited by the small sample size and the single-centre design of these RCTs; hence, further research seems warranted. Nonetheless, a recent consensus identified TFBs as one of the eight strategies identified as associated with “fast-track cardiac surgery” [64], suggesting that evidence is accumulating. We envisage that ability to perform TFBs could become a somewhat basic skill of cardiac anaesthesiologists in the near future.

Nonetheless, whenever implementing a medical approach, there is a need to gather evidence in support of it, and TFBs in cardiac surgery are no exception. Further, it is important to identify which TFBs are of greater value and which patients can benefit the most from TFBs in cardiac surgery. As mentioned, the vast majority of RCTs performed in this setting enrolled small sample sizes and have a single-centre design with difficult blinding inherent to the anaesthesiological technique. Hence, we believe that the best description of the direction of the results available is possibly provided by pooled evidence in the form of systematic reviews and meta-analyses. In this paragraph, we discuss the most recent ones with their main findings and possible limitations.

One of the most interesting meta-analyses published on this topic was conducted by Dost el al., where the authors selected only RCTs where a regional anaesthesia technique was performed with an ultrasound-guided approach in the setting of cardiac surgery. This meta-analysis included 15 RCTs with data on 849 patients (405 placebo), and with data on four techniques and a variable number of patients for each (PIP block n = 183; Deep PIP block n = 148; ESP block n = 93, and IPP block n = 20), confirming large heterogeneity in the included studies and ultimately an unbalanced sample. The authors reported a significantly reduced opioid consumption in the first 24 h after surgery. The best results were obtained by the ESP block, with a reduction of almost 23 morphine milligram equivalents (MME), though with a wide confidence interval [−34.3; −11.6], followed by Deep PIP and PIP (average reduction of −10.7 and −7.6 MME, respectively). Nonetheless, the authors conclude that the paucity of data available does not allow us to establish the best TFB and call for larger RCTs [65]. Another recent meta-analysis conducted by Greene et al. [66] investigated results of the ESP block in the context of midline sternotomy. The authors pooled 10 studies (nine RCTs and one prospective study, published in the years 2019–2023) conducted in both adult (n = 7) and paediatric (n = 3) cardiac surgery settings, though two RCTs in adults did not actually report data for the quantitative synthesis. In the included studies, the intervention group received the bilateral single-shot ESP block immediately before or just after induction of general anaesthesia. Both the intervention and the control group received patient-controlled analgesia. Interestingly, the intervention group in the included patients had a sample size variable from 20 to 53 patients. Though the average number of cardiac surgery procedures performed yearly may grossly vary, these numbers seem rather small, raising the question of possible patient selection and biases. Nonetheless, patients receiving the ESP block reported a significantly lower pain score at 4 h but not at 12 h. The postoperative use of opioids and the duration of mechanical ventilation were also significantly reduced but ICU and hospital stay were not affected. A subsequent meta-analysis conducted by Wang et al. [67] was published in the same journal but focused on the ultrasound-guided ESP block performed in adults and evaluated its analgesic effects after median sternotomy. The authors included eight RCTs with 543 patients and found that the ESP block effectively reduced pain scores immediately after extubation, and at 6 and 12 h after extubation, also reducing opioid consumption in the first 24 postoperative hours by roughly 35 MME, but the authors also highlighted the suboptimal evidence level of their results. In general, it seems that the ESP block, apart from targeting the ventral rami of the spinal nerves (and less commonly the dorsal rami) with the injection in the fascial plane anterior to the erector spinae group of muscles, may have some spread into the paravertebral space. Hence, it is not surprising that it could result in the best outcomes among TFBs. Few other meta-analyses have been conducted [68,69,70], but we believe it is not worth listing all of these studies as none of them can claim they are conclusive in their findings due to the limitations of the included studies and to the heterogeneity of the inclusion criteria. Apart from focusing on different types of TFBs, the main issues encountered in the meta-analyses currently published are as follows: (1) some included only RCTs whilst others extended the inclusion to non-randomized studies, (2) most of the included studies are single-centre, (3) the assessors were most frequently not blinded to the intervention, (4) in some meta-analyses the authors included both adult and paediatric patients, (5) the sample size of the studies included is rather small, (6) the timing of execution of the regional anaesthesia block may differ across studies, as does the dose of LA (with or without adjuvant). A further point of discussion is represented by the patient selection. Indeed, apart from factors related to the patients and their comorbidities, with the development of mini-invasive surgical approaches, anaesthesiologists will need to adequately select the appropriate regional anaesthesia technique.

Another potentially promising aspect that should be considered is the possibility that implementation of TFBs could reduce the risk of development of chronic post-sternotomy pain (CPSP), a condition that affects a non-negligible number of patients after cardiac surgery, with a variable incidence according to the length of follow-up [71,72,73]. For instance, Van Gulik et al. reported an incidence of 35% at 1 year after sternotomy with several risk factors identified by multivariate regression analysis (non-elective surgery, re-sternotomy, severe pain on 3rd postoperative day, and female gender) [73]. Subsequently, the same group of authors identified that intraoperative remifentanil dose was a predictor for CPSP in a dose-dependent manner [74]. In this regard, another literature gap remains. Indeed, future research should address whether the implementation of TFBs during the perioperative period may decrease the occurrence of CPSP; meanwhile, some emerging evidence supports the use of TFBs to control pain in patients suffering from CPSP [75,76].

From this summary, it appears that more evidence is needed. Interestingly, a recent meta-analysis by Kotani et al. with a focus on adult ICU patients reported that single-centre RCTs frequently do not translate into similar results when tested in multicentre RCTs [77]. Indeed, the authors identified 19 single-centre RCTs published in the three most influential medical journals (New England Journal of Medicine, JAMA, or Lancet) that had reported a significant decrease in mortality. Interestingly, 16 of these RCTs were subsequently followed by at least one multicentre RCT, and only one confirmed the finding of the previous single-centre RCT; in 14 cases the multicentre RCT was negative and in one case the multicentre RCT contradicted the findings of the single-centre RCT. We believe that this message is also important in the context of TFBs in cardiac surgery, where the efficacy of a regional anaesthesia technique in the hands of a skilled professional may not translate into effectiveness in the real-world scenario if tested in pragmatic trails. In this regard, adequate training and practice is of utmost importance to reach proficiency and maximize the results. Whilst it has been provocatively stated that a RCT is not always needed to determine the efficacy of an intervention [78], there are no real doubts as to the significant advantages of peripheral regional anaesthesia in the setting of cardiac surgery. Whether TFBs may not only aid short-term postoperative recovery but also boost the rehabilitation process and decrease the incidence of CPSP remains another subject where prospective data are needed. Nonetheless, the introduction of TFBs is still in its early days; adequately planned research is required to better understand which block (and solution of LA ± adjuvant) is the most appropriate according to the patient’s characteristics, type of intervention and surgical technique, and also the local institutional factors. Meanwhile, young cardiac anaesthesiologists should embrace the change and pursue training in this field, which represents a shift in perspective compared to previous years.

## 4. Conclusions

The latest revolution in the field of cardiac anaesthesia is probably the advent of TFBs, which provide several advantages in the context of multimodal analgesia and enhanced recovery after cardiac surgery. Several TFBs can be used according to the procedure, with tips and tricks gathered from experience and anatomical knowledge. However, the scientific evidence behind the implementation of TFBs in cardiac anaesthesia is certainly weak, with most studies designed as single-centre and enrolling small sample sizes. Hence, the resulting evidence is of low quality, and it remains unclear which cardiac surgery patients could benefit most from these regional anaesthesia techniques.

## Figures and Tables

**Table 1:** Thoracic wall fascial blocks and their main indications in the field of cardiac surgery. For each block, we also describe the nervous and anatomical targets, the injection site, and the suggested volume of local anaesthetic (LA), along with tips and tricks for the successful performance of the block.

Block	Indications in Cardiac Surgery Setting	Nervous and Anatomical Target, Injection Site, and Volume of LA	Tips and Tricks for Successful Block
**PIP**	Median sternotomy, mammary region pain after harvesting, sternal resynthesis/fractures, wire removal, sternal wound debridement, muscle flap reconstruction, ICD or pacemaker implant (with addition of SAP).	Anterior cutaneous branches of T2–T6 intercostal nerves. Medial Thoracic region. 2nd and 4th intercostal space. Bilaterally if sternotomy. 5–10 mL of LA.	Placing the tip of the needle on the dome of the rib allows an easier and more homogeneous diffusion of the LA, obtaining a wider dermatomal coverage.
**RSB**	Postoperative subxiphoid drainages pain management.	T7 to T11 intercostal nerves, and subcostal nerve (T12). Posterior rectus muscle sheath. 10–15 mL of LA.	The LA is injected between the muscle and its posterior sheath, not between the two lines appearing as binary shaped. The “double V” sign (opening of two fascial planes) is not visible. Caution is needed to avoid inferior epigastric artery injury.
**IPP**	Postoperative sternal analgesia, placement of anterior chest drains, post-traumatic chest injuries, ICD or pacemaker implant.	Lateral and medial pectoral nerves. Pectoral muscles. Anterior thoracic wall. Between pectoralis major and minor muscles. 10 mL of LA.	The lateral to medial approach allows a more comfortable position than the medial to lateral one. The pectoral branch of the thoraco-acromial artery is an important landmark to identify the inter-fascial plane. An effective block is realized when the ultrasound “double V” sign is visualized.
**PSP**	Pain management after thoracotomy in association with SAP block.	T2–T6 intercostal nerves lateral cutaneous branches. Lateral thoracic wall. Intercosto-brachial, thoracic longus and thoraco-dorsal nerves. 4th rib. 20 mL of LA.	Opposite decubitus to the surgical site; probe placed on the mid-axillary line. At level of 4th rib, the LA can be injected below the plane of serratus muscle realizing a deep SAP block or blocking the branches of intercostal nerves in the middle axillary line.
**SAP**	Pain management after mini-thoracotomies; continuous analgesia for thoracotomies; placement of chest drains, ICD, or pacemaker implant (with addition of IPP or PIP); pain due to rib fractures or thoracic trauma.	Lateral cutaneous branches of T2–T7 intercostal nerves. Lateral thoracic wall. 5th rib, Superficial or deep to serratus anterior muscle. 30 mL of LA.	Anatomical target of PECS II block is similar to deep SAP block in terms of dermatomal coverage and efficacy. If the probe is positioned along the mid-axillary line and the LA is deposited below the serratus muscle plane the technique could also be named “BRILMA”. Placing the needle tip on the rib avoids pleural puncture, achieving better diffusion of LA.
**ESP**	Cardiac surgery with mini-thoracotomy, open thoracotomy, rib fractures after mini-thoracotomy, chronic post thoracotomy pain syndrome.	Ventral rami of the spinal nerves. Dorsal rami when LA spreads into the paravertebral space. Posterior and lateral thoracic wall. Intercosto-brachial nerve. Between erector spinae muscle and T4–T5 transverse processes. 20–25 mL of LA.	The rib has a convex ultrasound shape, while the transverse process has a squared ultrasound shape. An effective ESP block is realized when the ultrasound erector spinae muscle lift is observed during the injection of LA

Pecto-Intercostal Plane (PIP), Rectus Sheath Block (RSB), Interpectoral Plane (IPP), Pecto-Serratus Plane (PSP), Serratus Anterior Plane (SAP) Erector Spinae Plane (ESP), Implantable Cardioverter Defibrillator (ICD).

## Abbreviations

The following abbreviations are used in this manuscript:

CPSP	Chronic Post-Sternotomy Pain
D-SAP	Deep Serratus Anterior Plane
EIM	External Intercostal Muscle
ERACS	Enhanced Recovery After Cardiac Surgery
ESM	Erector Spinae Muscle
ESP	Erector Spinae Plane
ICD	Implantable Cardioverter Defibrillator
IIM	Internal Intercostal Muscle
IM	Intercostal Muscle
IMA	Internal Mammary Artery
IMV	Internal Mammary Vein
IPP	Interpectoral Plane
ITP	Intertransverse Process
LA	Local Anaesthetic
LAM	Left Rectus Abdominis Muscle
LDM	Lastissimus Dorsi Muscle
LPN	Lateral Pectoral Nerve
MME	Morphine Milligram Equivalent
MPN	Medial Pectoral Nerve
N	Needle
PCA	Patient-Controlled Analgesia
IPP	Interpectorale Plane Block
PSP	Pectoserratus Plane Block
PIP	Pecto-Intercostal plane
PMM	Pectoralis Major Muscle
ppm	Pectoralis Minor Muscle
PSP	Pectoserratus Plane
RAM	Right Rectus Abdominis Muscle
RAMS	Rectus Abdominis Muscle Sheath
RCTs	Randomized Controlled Trials
RM	Rhomboid Muscle
RSB	Rectus Sheath Block
SAM	Serratus Anterior Muscle
SAP	Serratus Anterior Plane
SCTL	Superior Costotransverse Ligament
S-SAP	Superficial Serratus Anterior Plane
TAA	Thoraco-Acromial Artery
TFBs	Thoracic Fascial Blocks
TM	Trapezius Muscle
TP	Transversus Process
